# In Silico Analysis and Synthesis of Nafamostat Derivatives and Evaluation of Their Anti-SARS-CoV-2 Activity

**DOI:** 10.3390/v14020389

**Published:** 2022-02-14

**Authors:** Kazuhiro J. Fujimoto, Daniel C. F. Hobbs, Miki Umeda, Akihiro Nagata, Rie Yamaguchi, Yoshitaka Sato, Ayato Sato, Kohsuke Ohmatsu, Takashi Ooi, Takeshi Yanai, Hiroshi Kimura, Takayuki Murata

**Affiliations:** 1 Institute of Transformative Bio-Molecules (WPI-ITbM), Nagoya University, Nagoya 464-8601, Japan; 11akihiro15@gmail.com (A.N.); rie_yamaguchi@itbm.nagoya-u.ac.jp (R.Y.); ayato-sato@itbm.nagoya-u.ac.jp (A.S.); ohmatsu@chembio.nagoya-u.ac.jp (K.O.); tooi@chembio.nagoya-u.ac.jp (T.O.); yanait@chem.nagoya-u.ac.jp (T.Y.); 2 Department of Chemistry, Graduate School of Science, Nagoya University, Nagoya 464-8601, Japan; danhobbs22@gmail.com; 3 Department of Virology, Graduate School of Medicine, Nagoya University, Nagoya 466-8550, Japan; m.umeda@med.nagoya-u.ac.jp (M.U.); yssato@med.nagoya-u.ac.jp (Y.S.); hkimura@med.nagoya-u.ac.jp (H.K.); 4 Department of Molecular and Macromolecular Chemistry, Graduate School of Engineering, Nagoya University, Nagoya 464-8601, Japan; 5 PRESTO, Japan Science and Technology Agency (JST), Kawaguchi 332-0012, Japan; 6 Department of Virology and Parasitology, Fujita Health University School of Medicine, Toyoake 470-1192, Japan

**Keywords:** COVID-19, anti-SARS-CoV-2 agent, TMPRSS2, nafamostat, camostat

## Abstract

Inhibition of transmembrane serine protease 2 (TMPRSS2) is expected to block the spike protein-mediated fusion of severe acute respiratory syndrome coronavirus 2 (SARS-CoV-2). Nafamostat, a potent TMPRSS2 inhibitor as well as a candidate for anti-SARS-CoV-2 drug, possesses the same acyl substructure as camostat, but is known to have a greater antiviral effect. A unique aspect of the molecular binding of nafamostat has been recently reported to be the formation of a covalent bond between its acyl substructure and Ser441 in TMPRSS2. In this study, we investigated crucial elements that cause the difference in anti-SARS-CoV-2 activity of nafamostat and camostat. In silico analysis showed that Asp435 significantly contributes to the binding of nafamostat and camostat to TMPRSS2, while Glu299 interacts strongly only with nafamostat. The estimated binding affinity for each compound with TMPRSS2 was actually consistent with the higher activity of nafamostat; however, the evaluation of the newly synthesized nafamostat derivatives revealed that the predicted binding affinity did not correlate with their anti-SARS-CoV-2 activity measured by the cytopathic effect (CPE) inhibition assay. It was further shown that the substitution of the ester bond with amide bond in nafamostat resulted in significantly weakened anti-SARS-CoV-2 activity. These results strongly indicate that the ease of covalent bond formation with Ser441 in TMPRSS2 possibly plays a major role in the anti-SARS-CoV-2 effect of nafamostat and its derivatives.

## 1. Introduction

In December 2019, a new coronavirus (severe acute respiratory syndrome coronavirus 2; SARS-CoV-2 [1]) infectious disease (COVID-19) was confirmed in Wuhan, China, and spread around the world in a short period of time. COVID-19 has not only caused a public health problem, but its impact has also drastically changed our way of life, including economy and culture. The successful development of the COVID-19 vaccine [2,3,4,5] has given us great hope, and the required two doses of the vaccine are now being administered worldwide; however, as the titer of antibodies produced by the vaccine decreases over time [6,7], the need and plan for a third dose is being recommended in many countries. As of now (January 2022), the infection is still not under control and the path to the end of the pandemic is uncertain. There are several drugs that are already being used to treat COVID-19, such as the antiviral agent remdesivir [8,9] and the anti-inflammatory agent dexamethasone [10], which are already approved in many countries [11,12]. Several clinical trials for anti-SARS-CoV-2 drugs such as ivermectin (ClinicalTrials.gov; NCT04646109), Tocilizumab (NCT04320615 and NCT04356937) [13], and hydroxychloroquine (NCT04491994, NCT04334148, and NCT04332991) [14] failed to show superior efficacy to COVID-19 vaccines; although, many other trials still ongoing (details in https://clinicaltrials.gov/ct2/covid_view (accessed on: 10 January 2022)). In addition to the vaccine, the development of a number of COVID-19 therapeutic agents is essential for preventing the spread of the infection and maintaining social and economic activities.

SARS-CoV-2 is transmitted by spike protein (S protein) in the envelope, which binds to the angiotensin-converting enzyme 2 (ACE2) receptor on the target cell membrane and enters the cell [15]. The S protein is thought to be cleaved into S1 and S2 by the host protease furin [16,17]. S1 binds to the ACE2 receptor, and S2 is further cleaved by the cell-surface transmembrane serine protease 2 (TMPRSS2), resulting in membrane fusion [18].

This membrane fusion has been reported to be prevented by the TMPRSS2 inhibitor nafamostat, which is a drug for acute pancreatitis [18,19]. Based on these results, a clinical trial for nafamostat (*n* = 30) is ongoing (the Japan Registry of Clinical Trials; jRCTs031210183). The 50% effective concentration (EC_50_) of nafamostat was determined to be approximately 10 nM [19], showing 10-fold more potent anti-SARS-CoV-2 activity than the other TMPRSS2 inhibitor, camostat [19]. However, the reason why nafamostat has greater antiviral activity than camostat is unclear.

**Figure 1:**
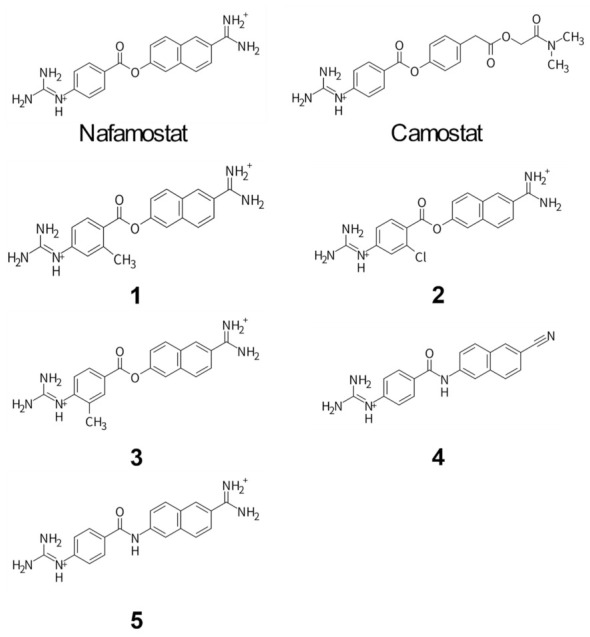
Chemical structures of nafamostat and its derivatives. Molecular docking simulations were performed using these compounds to obtain the binding conformations in the binding pocket of TMPRSS2. The binding affinities for these binding conformations were then evaluated using quantum chemical calculations.

A recent crystallographic study revealed that nafamostat does not continue to bind to the serine protease (SP) domain of its target protein, TMPRSS2, in its native form, but is converted to an acyl form with the hydrolysis product 4-guanidinobenzoic acid (GBA) covalently bound to Ser441 [20]. This may imply that the acylation of nafamostat with Ser441 is important for TMPRSS2 inhibition; although, most of the selective inhibitors approved for pharmaceutical use inhibit the enzyme activity by non-covalent binding. Interestingly, this chemically identical acyl form was also produced in the case of camostat, which was confirmed by the results of crystal structure analysis of camostat with other serine proteases, prostasin [21], enteropeptidase [22], and urokinase-type plasminogen activator (uPA) [23]. Therefore, the mechanism of TMPRSS2 inhibition seems to be the same for nafamostat and camostat. These facts led us to ask the following question: What accounts for the higher TMPRSS2 inhibitory activity of nafamostat than that of camostat? There are two possibilities: one is the difference in binding affinity to TMPRSS2, and the other is the difference in the ease of acylation with Ser441. As shown in Figure 1, the chemical structures of nafamostat and camostat are identical in the half up to the central ester bond moiety, but differ in the other half. This difference in chemical structure may cause the differences in binding affinity and/or acylation for TMPRSS2, resulting in the difference in antiviral effect. Although the reaction mechanism of nafamostat and camostat against TMPRSS2 has already been proposed [24], the binding affinities of these compounds and the effect of acylation have not been discussed yet. It is expected that elucidation of the molecular mechanism underlying the greater antiviral activity of nafamostat will lead to further optimization of the lead compound.

This study focuses on the anti-SARS-CoV-2 activities of nafamostat and its derivatives (compounds **1** to **5**). As mentioned above, the difference in the anti-SARS-CoV-2 activity between nafamostat and camostat is thought to involve their binding affinity and/or acylation to TMPRSS2. While it is difficult to directly analyze the process of acylation with Ser441 both experimentally and computationally, it is possible to computationally analyze the binding affinity to TMPRSS2 using quantum chemical calculations [25,26,27]. Therefore, we first evaluated the binding conformations and binding affinities of nafamostat and camostat to TMPRSS2 using molecular docking and quantum chemical calculations. These calculations revealed that specific amino acids (Glu299 and Asp435) strongly interact with nafamostat in the binding site of TMPRSS2, while one of these characteristic interactions is absent with camostat, suggesting that this interaction leads to the greater antiviral effect of nafamostat than camostat. These findings inspired us to design new nafamostat derivatives that may change the interactions with the specific amino acids. To further investigate whether acylation of the ligand is essential for the inhibition of TMPRSS2, we attempted to design nafamostat derivatives that are not readily acylated with Ser441. Thus, five nafamostat derivatives were actually synthesized. Subsequently, the cellular antiviral activity of these compounds was evaluated using SARS-CoV-2 infected cells. The results of the assay showed **2** and **3**, nafamostat derivatives with methyl or chloro groups, had 1.4 and 1.1 times greater antiviral activities than nafamostat, respectively. Moreover, **2** and **3** had no cytotoxicity against Calu-3 cells at concentrations up to 1 μM. The analysis also exhibited for the first time that the EC_50_ values differed approximately 1000-fold between the conditions with and without medium change. Furthermore, **4** and **5**, in which the ester bond moiety of nafamostat was replaced with an amide bond, showed very poor anti-SARS-CoV-2 activities, suggesting that acylation with Ser441 plays a crucial role in TMPRSS2 inhibition. Our findings here would be useful for further evaluation of nafamostat derivatives in clinical trials.

## 2. Materials and Methods

### 2.1. In Silico Analysis

To explore the binding conformations of ligands in the target protein TMPRSS2, we performed molecular docking simulations using the fitness learning-based artificial bee colony with proximity stimuli (F*l*ABCps) method [28] and the Glide method [29] of the Schrödinger software suit [30]. The atomic coordinates of TMPRSS2 for the crystal structure were obtained from the protein data bank (PDB) entry 7MEQ [20], which was used as the target protein for the ligands (i.e., camostat, nafamostat, and nafamostat derivatives) in the docking simulations. The computational conditions for the F*l*ABCps method were the same as for [25], while the default settings were used for the Glide method. The binding conformations of the ligands from the two methods were almost identical (RMSD < 1Å), confirming a negligible dependence on the computational method.

We analyzed the interaction between the ligand and each amino acid in TMPRSS2 for the ligand-binding conformations obtained by molecular docking. The interaction energy between the *i*-th amino acid and ligand was calculated with

(1) $$\Delta E_{i}=E_{ij}-\left(E_{i}+E_{j}\right),$$

where $E_{i}$ and $E_{j}$ denote total energies of molecule *i* (i.e., *i*-th amino acid) and molecule *j* (i.e., ligand), and $E_{ij}$ is the total energy of the molecular complex *ij*. The protein–ligand binding energy was obtained from

(2) $$E_{\mathrm{Bind}}=\sum_{i\in \mathrm{Protein}}\Delta E_{i},$$

where the summation runs over all amino acids in the protein. The *i*-th molecule in Equation (1) was created by breaking the peptide bond and capping it with a hydrogen atom, and their individual total energies were calculated using the second order of Møller–Plesset perturbation theory (MP2) [31] and the 6-31G(d) basis set. In the calculation of molecular complexes, the basis set superposition error (BSSE), which depends on the size of the basic functions used, makes it difficult to accurately estimate the interaction energy. We used the counterpoise method [32] to calculate the molecular energies corrected for the BSSE. These quantum chemical computations were carried out with the Gaussian16 program package [33].

### 2.2. Reagents

Nafamostat mesylate (N0959, 100 mg) and camostat mesylate (C2977, 100 mg) were purchased from Tokyo Chemical Industry (TCI, Tokyo, Japan).

### 2.3. Cell Lines and Virus Preparation

Vero (JCRB9013), VeroE6/TMPRSS2 (JCRB1819), and Calu-3 (HTB-55) cells were obtained from the Japanese Collection of Research Bioresources (JCRB) Cell Bank and American Type Culture Collection (ATCC), respectively. Vero and VeroE6/TMPRSS2 cells were cultured in Dulbecco’s modified Eagle’s medium (DMEM) (Nacalai-Tesque, Kyoto, Japan), supplemented with 5% fetal bovine serum (FBS) (Sigma-Aldrich, St. Louis, MO, USA) and penicillin-streptomycin (Sigma-Aldrich). Calu-3 cells were maintained in DMEM, supplemented with 10% FBS and the same antibiotics. SARS-CoV-2 (SARS-CoV-2/Hu/DP/Kng/19-020, a Wuhan strain isolated from a throat swab of a patient on the cruise ship Diamond Princess), was obtained from the Kanagawa Prefectural Institute of Public Health, Japan. The virus was propagated in Vero cells and titrated using VeroE6/TMPRSS2 cells as described previously [34].

### 2.4. Infection and MTS Assays

Calu-3 cells were seeded on 96-well plates one day before infection. Before infection, the media were changed to those containing various concentrations of inhibitors and incubated for 1 h. The media were removed and added with those containing the same concentrations of inhibitors and SARS-CoV-2 (at a multiplicity of infection of 0.01). For “medium change” group, the media containing the virus were removed after 30 min and added with fresh media containing the same concentrations of inhibitors. The media were changed at 1 and 2 days after infection to the fresh media containing the same dose of inhibitors. For “no medium change” group, the cells were pre-treated for 1 h with various concentrations of inhibitors, added with those containing the same concentrations of inhibitors (at the same dose) and SARS-CoV-2, but the media were not changed after infection. After 5 days, CPE-positive wells were examined and EC_50_ was determined by calculating approximated sigmoidal equation (four parameter logistic curve) using ImageJ 1.53k. Two independent experiments were performed for each group and the ratio of CPE positivity for each concentration was determined by counting CPE-positive wells among 12 independent wells. MTS assays were carried out by using CellTiter 96 AQueous One Solution Cell Proliferation Assay kit (Promega, Madison, WI, USA) and Multiskan FC microplate reader (Thermo Fisher Scientific, Waltham, MA, USA) according to the manufacturer’s instructions.

## 3. Results

### 3.1. In Silico Analysis of Nafamostat Binding to TMPRSS2

In this section, we first analyzed the binding conformation of nafamostat to TMPRSS2 and its interaction with amino acids constituting TMPRSS2. Then, the same analysis was performed for camostat, and the results of nafamostat and camostat were compared. It is important to note that the crystal structure of TMPRSS2 revealed that GBA, one of the hydrolysis products of nafamostat and camostat, is covalently bound to Ser441 [20], but it is unclear how nafamostat and camostat were arranged in the binding pocket of TMPRSS2 prior to the covalent bond formation to Ser441.

**Figure 2:**
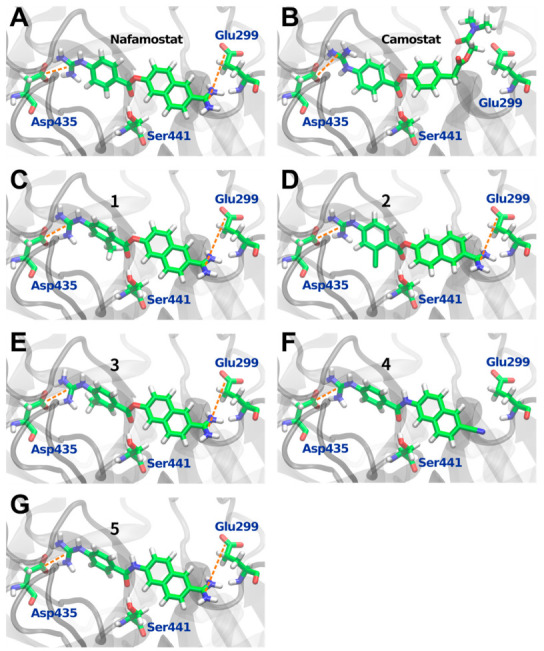
Binding structures of (**A**) nafamostat, (**B**) camostat, and (**C**–**G**) nafamostat derivatives (compounds **1** to **5**) at the binding site of TMPRSS2. The gray ribbon represents the backbone structure of TMPRSS2. In (**A**), the cationic guanidino group of nafamostat is in close proximity to the anionic carboxyl group of Asp435, resulting in a strong electrostatic attraction. In addition, the cationic amidino group of nafamostat is close to the anionic carboxyl group of Glu299, resulting in a strong electrostatic attraction. In (**B**), strong electrostatic attraction with Asp435 occurs in camostat as well as in nafamostat, but the interaction with Glu299 is weaker because the cationic amidino group is not present in camostat. The orange dotted lines represent strong electrostatic attractions.

In order to investigate the binding conformation of nafamostat to TMPRSS2, a molecular docking simulation was performed. As shown in Figure 2A, the nafamostat structure obtained with the docking simulation was found to fit linearly into the binding pocket of TMPRSS2 while it was slightly twisted at the molecular center. The orientation of the guanidino group of the nafamostat was toward Asp435, which is similar to the orientation of the guanidino group of GBA observed in the crystal structure [20]. In addition, the ester bond moiety at the center of nafamostat was located near Ser441, which is a position where GBA is covalently bound to Ser441, as observed in the crystal structure.

**Figure 3:**
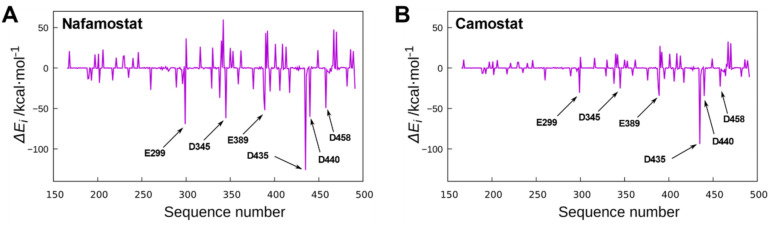
Contribution of amino acids to the binding energy of (**A**) nafamostat and (**B**) camostat. The positive and negative peaks represent repulsive and attractive interactions, respectively. The respective interaction energies were calculated using Equation (1). The arrows indicate the amino acids that contribute significantly to the binding energy. In both (**A**,**B**), the strongest attractive interaction arises from Asp435, while a strong attractive interaction with Glu299 is observed in (**A**), but not in (**B**).

To further investigate the binding structure of nafamostat, the interaction energies between nafamostat and each amino acid were estimated using quantum chemical calculations at the MP2 level of theory [31,33]. The interaction energy with each amino acid was calculated using Equation (1) [25] (see Material and Methods Section for details). The calculated interaction energies are shown in Figure 3A. It should be noted that the positive and negative values in the interaction energy represent the contributions of repulsion and attraction, respectively. The results show the largest negative peak corresponding to the strongest interactions with Asp435. As mentioned above, the guanidino group of nafamostat is located in proximity to Asp435. Therefore, the large negative peak of the interaction energy appears due to the strong electrostatic interaction between the positively charged guanidino group of nafamostat and the negatively charged carboxyl group of Asp435. A similar explanation applies to the second largest negative peak corresponding to the interaction with Glu299. The strong electrostatic interaction occurs because the positively charged amidino group of nafamostat is in close proximity to the negatively charged carboxyl group of Glu299. The crystal structure of TMPRSS2 reveals that the guanidino group of GBA faces Asp435 [20], but it remained unclear with which amino acid the other half of the nafamostat interacts strongly before forming a covalent bond with Ser441. The present analysis suggests that nafamostat may interact strongly not only with Asp435 but also with Glu299.

The same analysis was performed for camostat, which is also a drug for acute pancreatitis and a known TMPRSS2 inhibitor [35]. The binding conformation and interaction energies of camostat are shown in Figure 2B and Figure 3B, respectively. The results show that, as in the case of nafamostat, the largest negative peak in Figure 3B corresponds to the interaction of camostat with Asp435. On the other hand, the interaction between camostat and Glu299 was found to be small, which is in contrast to that of nafamostat. These results are well explained by the binding conformation of camostat shown in Figure 2B. The structure of camostat fits into the binding pocket of TMPRSS2 with the guanidino group facing the carboxyl group of Asp435, resulting in the strong electrostatic interaction with Asp435. The form of this interaction is almost the same as that of nafamostat. On the other hand, camostat has no positively charged functional groups near Glu299, which results in a weak interaction with Glu299. Thus, one less cationic functional group in camostat makes the interaction of camostat with amino acids weaker than that of nafamostat. The total interaction energy with each amino acid (i.e., the protein–ligand binding energy) indicates that nafamostat (−178.22 kcal/mol) binds to TMPRSS2 approximately 50 kcal/mol more strongly than camostat (−128.84 kcal/mol). These results strongly suggest that the higher binding affinity of nafamostat to TMPRSS2 prior to its acylation with Ser441 is responsible for the greater anti-SARS-CoV-2 effect of nafamostat than camostat.

### 3.2. Design and Synthesis of Nafamostat Derivatives

The findings from the in silico analysis led us to design new nafamostat derivatives to modify the interactions of nafamostat with Glu299 and Asp435. First, we hypothesized that changing the orientation of the guanidino group of nafamostat would enhance its electrostatic interaction with Asp435. To this end, we designed three types of nafamostat derivatives by introducing a methyl or chloro group to the benzene ring to facilitate the rotation of the guanidino group (compound **1**, **2**, and **3**). In addition to these, we considered the effect of acylation on TMPRSS2 inhibition. The ester bond moiety of nafamostat is susceptible to hydrolysis and is considered to be less stable. Therefore, we designed a nafamostat derivative in which the cationic functional groups on both sides were not modified, but only the central ester bond was changed to an amide bond (compound **5**). It has been shown experimentally that proteases such as trypsin and papain hydrolyze amide bond more slowly than ester bonds [36,37]. Therefore, the substitution to an amide bond is expected to make acylation with Ser441 more difficult, even though the cationic functional groups on both sides maintain strong interactions with Glu299 and Asp435. Furthermore, a design with a weaker interaction with Glu299 was considered. As can be seen from Figure 2A, if we remove the cationic amidino group from the naphthalene ring of nafamostat, the electrostatic interaction with Glu299 is expected to be weaker. Therefore, we designed a nafamostat derivative in which the cationic amidino group on the naphthalene ring was replaced by a neutral cyano group (compound **4**). Here, the ester bond was also changed to an amide bond.

In silico analysis of the newly designed nafamostat derivatives was carried out in the same manner as that performed for nafamostat and camostat. Their binding conformations are shown in Figure 2, and the protein–ligand binding energies are summarized in Table 1. It should be noted that a larger negative binding energy represents a stronger attractive interaction between the ligand and TMPRSS2. The results showed that two compounds, **5** (−179.65 kcal/mol) and **3** (−178.94 kcal/mol), bind to TMPRSS2 more strongly than nafamostat (−178.22 kcal/mol). Compound **5** interacted with TMPRSS2 most strongly among the designed nafamostat derivatives. From the binding structure of **5**, it was confirmed that the interaction with Glu299 and Asp435 was maintained even when the ester bond was replaced with an amide bond. However, the orientation of the guanidino group was slightly different to that of nafamostat, resulting in increased electrostatic attraction to Asp435. The second strongest interaction was found in **3**, where its binding energy was more negative than that of nafamostat, indicating that it binds to TMPRSS2 more strongly than nafamostat. This is because the methyl group introduced in **3** changed the orientation of the guanidino group and strengthened its attraction to Ser436. On the other hand, the predicted binding energies showed that **1** (−172.38 kcal/mol), **2** (−174.16 kcal/mol), and **4** (−127.92 kcal/mol) have a weaker binding interaction to TMPRSS2 than nafamostat. These are mainly due to steric hindrance between **1** and Thr459, decreased electrostatic attraction between **2** and Asp440, and decreased electrostatic attraction between **4** and Glu299. In particular, the interaction of **4** with TMPRSS2 was significantly weakened by the substitution of the cationic amidino group with a neutral cyano group. Therefore, the importance of the cationic amidino group in nafamostat in the binding affinity with TMPRSS2 is greatly reinforced. In addition, the comparison of the results between **1** and **3** clearly showed that the difference in the position of the methyl group introduced had a significant effect on the interaction with TMPRSS2.

### 3.3. Antiviral Effects of Nafamostat Derivatives against SARS-CoV-2

The results of the in silico analysis suggested two things: firstly, the potential of the newly designed nafamostat derivatives as anti-SARS-CoV-2 agents beyond nafamostat, and secondly, the effectiveness of the nafamostat derivatives in investigating the mechanism of the anti-SARS-CoV-2 effect. In order to verify the two suggestions experimentally, we attempted to, and succeeded in, synthesizing and purifying all five derivatives, as described in detail in the Supplementary Materials.

**Figure 4:**
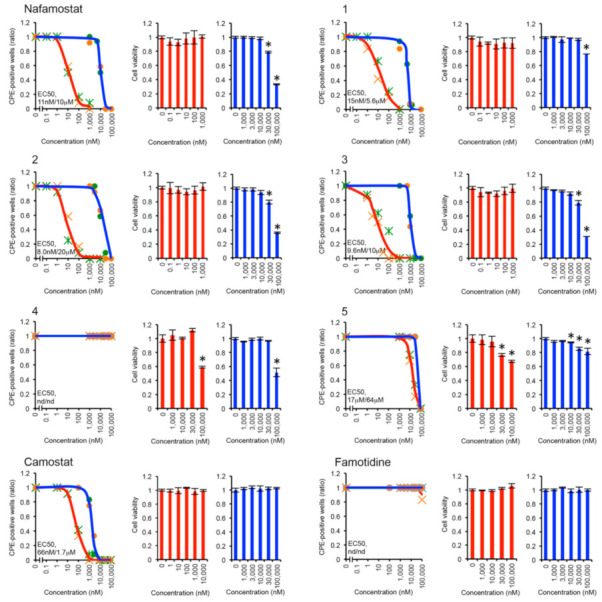
Antiviral effect of possible TMPRSS2 inhibitors (line charts). For “medium change (red lines)” condition, Calu-3 cells on 96-well plates were pre-treated with various concentrations of inhibitors for 1 h, and then infected with SARS-CoV-2 at low multiplicity of infection (MOI) for 30 min, followed by media change. Note that inhibitors were continuously added until the end. For “no medium change condition (blue lines)”, Calu-3 cells were treated likewise, except that media were not changed after infection. After 5 days, numbers of cytopathic effect (CPE)-positive wells were counted and EC50 was determined. Two independent experiments were performed for each condition and the ratios of CPE-positive wells are plotted as green or orange markers. MTS assay was also carried out to evaluate the effect of inhibitors on cell viability (bar charts). For this purpose, cells were treated in the similar fashion under “medium change (red bars)” and “no medium change (blue bars)” conditions, but the virus was not inoculated. Three independent experiments were performed for each condition, and the mean ± SD are shown. An asterisk indicates *p* < 0.005 by Student’s *t*-test.

Next, the anti-SARS-CoV-2 effects of the nafamostat derivatives were examined by the cytopathic effect (CPE) inhibition assay using Calu-3 cells infected with SARS-CoV-2 [19]. The results of EC_50_ for each ligand are illustrated in Figure 4. Here, nafamostat and camostat were also evaluated for comparison. There are also reports that the use of famotidine is likely to be clinically beneficial in reducing mortality in patients with COVID-19 [38], so that this assay was also applied to famotidine. It should be noted that the assays were performed in two ways: one under the condition with medium change and the other without medium change. To the best of our knowledge, no such comparison of the two assays has been reported before. In both assays, all compounds except **4** and famotidine inhibited CPE caused by SARS-CoV-2 infection in a dose-dependent manner. However, the EC_50_ values determined under the condition with medium change were approximately three orders of magnitude smaller than those without medium change. These results indicate that continuous administration of fresh compounds to the infected cells enhances the anti-SARS-CoV-2 effect. It has been reported that nafamostat has a short half-life in blood and is currently used as continuous intravenous drip in the treatment of pancreatitis [39]. Considering these facts and our results, it can be inferred that continuous intravenous drip is also appropriate for the treatment of COVID-19. Since the assay with medium change shows greater antiviral effect, the results measured with medium change are discussed below. 

The EC_50_ of nafamostat and camostat were determined to be 11 and 66 nM, respectively. These values are comparable to those of previous studies [19], confirming that nafamostat has a greater anti-SARS-CoV-2 effect than camostat. However, in the case of the assay without medium change, camostat showed a greater antiviral effect than nafamostat. Although it might be interesting to explore the reason why, we did not pursue it further in this paper.

The EC_50_ of famotidine could not be determined by this assay. Although famotidine may be clinically beneficial for the treatment of COVID-19, our results indicate that famotidine is not effective, at least against SARS-CoV-2 infected cells, consistent with a previous report [40].

In the present results, **2** and **3** showed the lowest and second lowest EC_50_, respectively. Their EC_50_ values were 8.0 and 9.6 nM, indicating that they have 1.4 and 1.1 times more potent antiviral activities than nafamostat (11 nM), respectively. The third lowest EC_50_ among the nafamostat derivatives was observed for **1** (15 nM). However, it was approximately 0.7-fold lower antiviral activity than nafamostat. The EC_50_ of **5** was 17 μM, and the EC_50_ of **4** could not be determined. Therefore, the antiviral effects of both **4** and **5** were found to be very poor. This may be due to the amide bond introduced into **4** and **5**, which makes it difficult to acylate with Ser441.

The experimental results obtained here are compared with the results of the in silico analysis. The results of the CPE inhibition assay showed that **3** had a greater antiviral effect than nafamostat, which was consistent with the result of the in silico analysis showing that **3** had a higher binding affinity for TMPRSS2 than nafamostat. In addition, the computational result for **4**, which showed the lowest binding affinity, was consistent with the experimental result of the very poor antiviral effect of **4**. Furthermore, the computational result showing a lower binding affinity of **1** than that of nafamostat was in good agreement with the experimental result showing a less antiviral effect of **1** than that of nafamostat. In contrast, the results of in silico analysis of **2**, which showed a lower binding affinity than nafamostat, was not supported by the experimental result evincing a greater antiviral effect of **2** than nafamostat. The largest discrepancy between the CPE inhibition assay and the in silico analysis was observed for **5**. Although **5** showed the highest binding affinity in the computation, the experimental results showed its very poor antiviral effect. A similar discrepancy was observed in the comparison between **4** and camostat; the difference in the antiviral effect between **4** and camostat was much larger than the extent of the difference in their binding affinities. The common feature between **4** and **5** is the introduction of an amide bond, which would make the acylation with Ser441 less likely than the ester bond [36,37], thereby preventing the inhibition of TMPRSS2. This result suggests that acylation with Ser441 is crucial for the expression of anti-SARS-CoV-2 activity of nafamostat.

To evaluate the cytotoxicity of the compounds used in the CPE inhibition assay on Calu-3 cells, we also performed the MTS assay. The cytotoxic effect was examined by measuring the viability of Calu-3 cells in the absence or presence of the compounds at different concentrations. The results are displayed in Figure 4. It should be noted that the maximum concentrations of the added compounds were varied according to the results of the CPE inhibition assay. For nafamostat, the viability of Calu-3 cells remained relatively constant up to 1 µM under the condition with medium change and up to 10 µM under the condition without medium change. Similar results were obtained for nafamostat derivatives. For **2** and **3**, which had lower EC_50_ than nafamostat, the viability of Calu-3 cells remained relatively constant up to 1 μM under the condition with medium change and up to 10 μM under the condition without medium change. These results indicate that nafamostat and its derivatives are not toxic to Calu-3 cells, even at concentrations at least 100 times higher than their EC_50_ concentrations. For camostat, the viability of Calu-3 cells remained relatively constant up to 10 μM under the condition with medium change and up to 100 μM without medium change. This indicates that camostat is even less cytotoxic than nafamostat.

## 4. Discussion

In this study, we investigated the difference in anti-SARS-CoV-2 activity between nafamostat and camostat in terms of binding affinity to TMPRSS2, and reported for the first time that the nafamostat derivatives **2** and **3**, which were designed based on the computational analysis of ligand–amino acid interactions, had greater anti-SARS-CoV-2 activities than nafamostat in infected cells. In silico analysis using molecular docking and quantum chemical calculations revealed that the cationic amidino group present in nafamostat, but not in camostat, induces a strong electrostatic interaction with Glu299 of TMPRSS2, resulting in a higher binding affinity of nafamostat to TMPRSS2 than that of camostat. Furthermore, by taking into account the interaction with amino acids, five new nafamostat derivatives were designed and their binding affinities to TMPRSS2 were evaluated by the in silico approach. The results showed that **3** and **5** had higher binding affinity than nafamostat (Table 1). Based on these results, five nafamostat derivatives were synthesized and their anti-SARS-CoV-2 activities were experimentally evaluated. The results showed that **2** and **3** inhibited the infection of SARS-CoV-2 in a dose-dependent manner at concentrations ranging from 0.1 to 1000 nM (Figure 4). The EC_50_ values of **2** and **3** were determined to be 8.0 and 9.6 nM, respectively, while that of nafamostat was 11 nM (Figure 4). These results indicate that the anti-SARS-CoV-2 activities of **2** and **3** are 1.4 and 1.1 times greater than that of nafamostat, respectively. We also performed the MTS assay to evaluate the cytotoxicity of **2** and **3** in vitro and found that **2** and **3** were not toxic to Calu-3 cells up to 1 μM (Figure 4). Although this study examined the anti-SARS-CoV-2 effect of the nafamostat derivatives using the Wuhan strain, we speculate that these compounds are equally effective against other variants such as the Delta and Omicron strains because the amino acid sequence around the TMPRSS2 cleavage site of the S protein is also conserved in SARS-CoV-2 variants (Supplemental Figure S1).

In this study, we evaluated the effect of ligand binding affinity to TMPRSS2 and acylation with TMPRSS2 on anti-SARS-CoV-2 activity. In silico analysis showed that **5** had the highest binding affinity among the compounds evaluated, whereas the poor anti-SARS-CoV-2 activity of **5** was confirmed by the CPE inhibition assay. Compound **5** is a nafamostat derivative in which the ester bond is replaced by an amide bond, while the cationic functional groups at both sides are maintained. The effect of these cationic functional groups was predicted to induce strong electrostatic interactions with Glu299 and Asp435 in TMPRSS2. However, its poor antiviral effect was confirmed by the assay. This is probably due to the amide bond introduced into **5**, which makes acylation with Ser441 less likely to occur. A similar explanation would be valid for **4**. The experimental results showed that **4** had a much poorer antiviral effect than camostat, even though the binding affinities of camostat and **4** were comparable in in silico analysis. This would also be due to the inhibition of acylation with Ser441 by the amide bond introduced into **4**. These findings suggest that the acylation with Ser441 in TMPRSS2 plays a crucial role in the anti-SARS-CoV-2 activity of nafamostat. This study demonstrated that one of the reasons for the greater anti-SARS-CoV-2 activity of nafamostat than camostat is the higher binding affinity of nafamostat to TMPRSS2. However, this study did not provide a direct comparison of the effect of acylation between nafamostat and camostat. The higher binding affinity of nafamostat over camostat may influence nafamostat to be more readily acylated to Ser441. This analysis will be the subject of future research. The findings and approach taken in this study provide a basis for the development of new types of anti-SARV-CoV-2 agents and tools for future research.

## Figures and Tables

**Table 1:** TMPRSS2-ligand binding energies (kcal/mol).

Compound	*E*_Bind_^1^
Nafamostat	–178.22
Camostat	–128.84
**1**	–172.38
**2**	–174.16
**3**	–178.94
**4**	–127.92
**5**	–179.65

^1^ The binding energies were calculated with Equation (2).

## Data Availability

Not applicable.

## Supplementary Materials

The following supporting information can be downloaded at: https://www.mdpi.com/article/10.3390/v14020389/s1, Figure S1: Alignment of the S protein sequence of SARS-CoV-2 variants, General Information for the Synthesis of Nafamostat Analogues.
