# Supplementary material for: In Silico Analysis and Synthesis of Nafamostat Derivatives and Evaluation of Their Anti-SARS-CoV-2 Activity
Source: Viruses. 2022 Feb 14;14(2):389. doi: 10.3390/v14020389 (PMC8876814; doi:10.3390/v14020389)
Supplement: Supplementary file 1 [file viruses-14-00389-s001.zip › viruses-1573140-supplementary.pdf]

In silico analysis and synthesis of nafamostat derivatives and evaluation of their anti-SARS-CoV-2 activity

Kazuhiro J. Fujimoto[1, 2, *], Daniel C. F. Hobbs[2], Miki Umeda[3], Akihiro Nagata[1, 4], Rie Yamaguchi[1, 4], Yoshitaka Sato[3, 5], Ayato Sato[1], Kohsuke Ohmatsu[1, 4], Takashi Ooi[1, 4], Takeshi Yanai[1, 2], Hiroshi Kimura[3], and Takayuki Murata[3, 6, *]

[1] *Institute of Transformative Bio-Molecules (WPI-ITbM), Nagoya University, Nagoya, Japan*

[2] *Department of Chemistry, Graduate School of Science, Nagoya University, Nagoya, Japan*

[3] *Department of Virology, Nagoya University Graduate School of Medicine, Nagoya, Japan*

[4] *Department of Molecular and Macromolecular Chemistry, Graduate School of Engineering, Nagoya University, Nagoya, Japan*

[5] *PRESTO, Japan Science and Technology Agency (JST), Kawaguchi, Japan*

[6] *Department of Virology and Parasitology, Fujita Health University School of Medicine, Toyoake, Japan*

* Correspondence:

fujimotok@chem.nagoya-u.ac.jp (K.J. Fujimoto),

tmurata@fujita-hu.ac.jp (T. Murata).

Pango lineage			S2'		
**B**	(Wuhan-Hu-1)	796	DFGGFNFSQILPDPSKPSKR	SFIEDLLFNKVTLADAGFIK	832
**B.1.1.7**	(Alpha)	793	DFGGFNFSQILPDPSKPSKR	SFIEDLLFNKVTLADAGFIK	832
**B.1.351**	(Beta)	793	DFGGFNFSQILPDPSKPSKR	SFIEDLLFNKVTLADAGFIK	833
**P.1**	(Gamma)	796	DFGGFNFSQILPDPSKPSKR	SFIEDLLFNKVTLADAGFIK	828
**B.1.617.2**	(Delta)	794	DFGGFNFSQILPDPSKPSKR	SFIEDLLFNKVTLADAGFIK	832
**BA.1**	(Omicron)	793	YFGGFNFSQILPDPSKPSKR	SFIEDLLFNKVTLADAGFIK	835
**C.37**	(Lambda)	789	DFGGFNFSQILPDPSKPSKR	SFIEDLLFNKVTLADAGFIK	835
**B.1.621**	(Mu)	796	DFGGFNFSQILPDPSKPSKR	SFIEDLLFNKVTLADAGFIK	835
			*******************	********************	

Figure S1. Alignment of the S protein sequence of SARS-CoV-2 variants. All variants listed as variants of concern (VOCs) and variants of interest (VOIs) by the WHO (As of February 2022, https://www.who.int/en/activities/tracking-SARS-CoV-2-variants/) possess a conserved TMPRSS2-cleavage site (red line). The NCBI accession numbers for S protein are as follows: B (YP_009724390.1), B.1.1.7 (UKO09266.1), B.1.351 (UJZ23686.1), P.1 (UKC35055.1), B.1.617.2 (UKP93498.1), BA.1 (UKP94254.1), C.37 (UKC50930.1), and B.1.621 (UJU97158.1).

General Information for the Synthesis of Nafamostat Analogues

^{1}H NMR spectra were recorded on a JEOL JNM-ECS400 (400 MHz) and JEOL JNM-ECA600II (600 MHz) spectrometer. Chemical shifts are reported in ppm from the tetramethylsilane (0.0 ppm) resonance as the internal standard (CD_3OD or DMSO-d_6). Data are reported as follows: chemical shift, integration, multiplicity (s = singlet, d = doublet, t = triplet, q = quartet, m = multiplet, and br = broad) and coupling constants (Hz). ^{13}C NMR spectra were recorded on a JEOL JNM-ECA600II (151 MHz) spectrometer with complete proton decoupling. Chemical shifts are reported in ppm from the solvent resonance as the internal standard (CD_3OD; 49.15 ppm, DMSO-d_6; 39.52 ppm). The high-resolution mass spectra were measured on Thermo Fisher Scientific Exactive Plus (ESI). Flash column chromatography was performed on Silica gel 60 N (spherical, neutral, 40~50μm; Kanto Chemical Co., Inc.).

Air- and moisture-sensitive reactions were performed under an atmosphere of argon (Ar) in dried glassware. 1,2-Dichloroethane (DCE) and acetonitrile (MeCN) were supplied from Kanto Chemical Co., Inc. as "Dehydrated" and further purified by both A2 alumina and Q5 reactant using a GlassContour solvent dispensing system. Other simple chemicals were purchased and used as such. Nafamostat analogues were prepared by a modified literature procedure.[1]

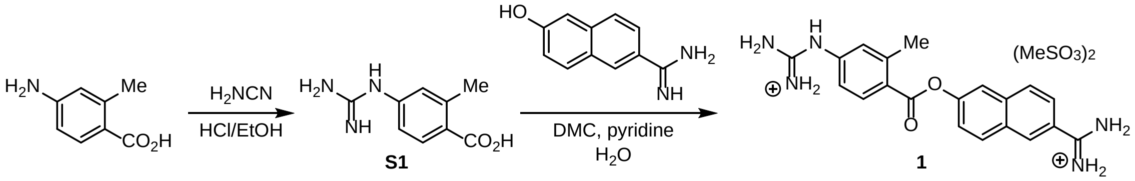

A mixture of 4-amino-2methylbenzoic acid (755 mg, 5 mmol) and cyanamide (50 w% aqueous solution, 4.2 mL, 50 mmol) in 6N aqueous solution of HCl (5 mL) and ethanol (20 mL) was stirred at room temperature for 14 h. The reaction mixture was concentrated in vacuo and the resulting crude material was washed with $CHCl_3$ and methanol. The material thus obtained was used for the next step without further purification. **S1**: ^{1}H NMR (400 MHz, CD_3OD) δ 8.01 (1H, d, J = 8.0 Hz), 7.46 (1H, brs), 7.20-7.15 (2H, m), 2.61 (3H, s).

To a mixture of **S1** (19.3 mg), 6-amidino-2-naphthol methanesulfonate (28.2 mg, 0.1 mmol), and 2-chloro-1,3-dimethylimidazolinium chloride (DMC, 50.7 mg, 0.3 mmol) in H_2O (1.25 mL) was added pyridine (48 μL, 0.6 mmol) at room temperature. After 4 h with stirring, 10% aqueous solution of $NaHCO_3$ was added to the reaction mixture. The precipitate was collected, washed with 1:1 mixture of H_2O and acetone, and then dissolved into acetone. To this solution was added an ion-exchange resin (amberlyst A-26, $MeSO_3^-$ form, 3.2 g), and the resulting mixture was

[1] (a) Sagi, K.; Fujita, K.; Sugiki, M.; Takahashi, M.; Takehana, S.; Tashiro, K.; Kayahara, T.; Yamanashi, M.; Fukuda, Y.; Oono, S.; Okajima, A.; Iwata, S.; Shoji, M.; Sakurai, K. *Bioorg. Med. Chem.* **2005**, *13*, 1487–1496. (b) Aoyama, T.; Okutome, T.; Nakayama, T.; Yaegashi, T.; Matsui, R.; Nunomura, S.; Kurumi, M.; Sakurai, Y.; Fujii, S. *Chem. Pharm. Bull.* **1985**, *33*, 1458−1471.

sonicated for 30 min. Filtration with the aid of 1:1 mixture of H_2O and acetone gave the product **1**.

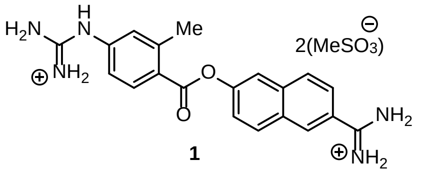

1

1: ^{1}H NMR (400 MHz, DMSO-d_6) δ 9.45 (2H, br), 9.10 (2H, br), 8.55 (1H, s), 8.24-8.17 (3H, m), 8.00 (1H, s), 7.87 (1H, d, J = 9.2 Hz), 7.65 (1H, d, J = 9.2 Hz), 7.57 (2H, br), 7.25-7.22 (2H, m), 2.62 (3H, s), 2.31 (6H, s); ^{13}C NMR (151 MHz, DMSO-d_6) δ 165.6, 164.6, 158.2, 155.2, 150.5, 142.5, 135.7, 132.8, 131.1, 130.9, 129.8, 129.5, 128.4, 125.7, 125.5, 124.6, 123.6, 120.4, 119.1, 40.6, 29.6; HRMS (ESI) Calcd for $C_{20}H_{20}N_5O_2^+$ ([M−H(OSO$_2$Me)$_2$]$^+$) 362.1612. Found 362.1606.

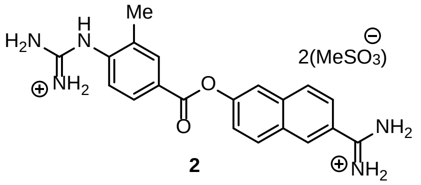

2

2: ^{1}H NMR (600 MHz, DMSO-d_6) δ 9.46 (2H, br), 9.12 (2H, br), 8.56 (1H, s), 8.23 (1H, d, J = 9.0 Hz), 8.19 (1H, d, J = 8.4 Hz), 8.15 (1H, s), 8.07 (1H, d, J = 8.4 Hz), 8.01 (1H, s), 7.88 (1H, d, J = 8.4 Hz), 7.65 (1H, d, J = 9.0 Hz), 7.45 (1H, d, J (1H, d, J = 8.4 Hz), 7.39 (2H, br), 3.26 (3H, s), 2.26 (6H, s); ^{13}C NMR (151 MHz, DMSO-d_6) δ 165.5, 164.2, 155.7, 150.4, 135.7, 135.1, 132.7, 131.0, 129.8, 129.5, 128.9, 128.5, 127.1, 125.6, 124.6, 123.4, 119.0, 40.4, 29.6, two peaks for aromatic carbons were not found probably due to the overlapping; HRMS (ESI) Calcd for $C_{20}H_{20}N_5O_2^+$ ([M−H(OSO$_2$Me)$_2$]$^+$) 362.1612. Found 362.1611.

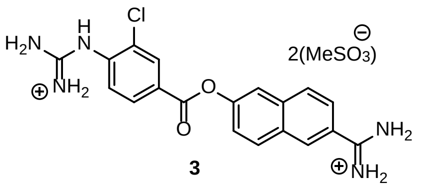

3

3: ^{1}H NMR (400 MHz, DMSO-d_6) δ 8.43 (1H, s), 8.10 (1H, d, J = 9.2 Hz), 8.05 (1H, d, J = 8.4 Hz), 8.01 (2H, s), 7.83 (1H, s), 7.50 (1H, d, J = 9.2 Hz), 7.05 (2H, br), 6.96 (1H, s), 6.90 (1H, d, J = 8.4 Hz), 2.12 (6H, s); ^{13}C NMR (151 MHz, DMSO-d_6) δ 163.1, 154.7, 149.2, 134.7, 134.2, 133.5, 130.2(4), 130.2(0), 127.3, 126.2, 125.2, 124.5, 122.5, 121.3, 118.6, 116.2, 40.1, three peaks for aromatic carbons were not found probably due to the overlapping; HRMS (ESI) Calcd for $C_{19}H_{17}Cl_1N_5O_2^+$ ([M−H(OSO$_2$Me)$_2$]$^+$) 382.1065. Found 382.1068.

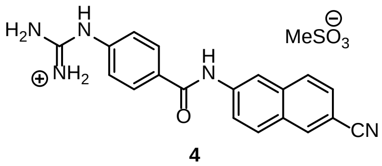

4

4: ^{1}H NMR (400 MHz, CD_3OD) δ 8.48 (1H, s), 8.32 (1H, s), 8.00-7.95 (4H, m), 7.91 (1H, d, J = 9.0 Hz), 7.64 (1H, d, J = 8.4 Hz), 7.18-7.16 (2H, m), 2.76 (3H, s); ^{13}C NMR (151 MHz, CD_3OD) δ 168.7, 156.9, 140.9, 137.0, 135.0, 130.8, 130.2(9), 130.2(6), 130.1, 127.9, 124.9, 123.6, 120.3, 118.0, 109.0, 39.7, two peaks for aromatic carbons were not found probably due to the overlapping; HRMS (ESI) Calcd for $C_{19}H_{16}N_5O_1^+$ ([M−(OSO$_2$Me)]$^+$) 330.1349. Found 330.1351.

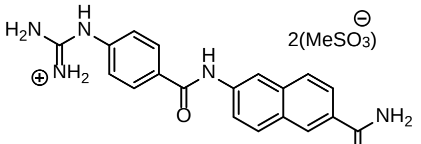

5

5: ^{1}H NMR (400 MHz, CD_3OD) δ 8.56 (1H, s), 8.30 (1H, s), 8.18 (2H, d, J = 7.8 Hz), 8.12-8.05 (3H, m), 7.95 (1H, d, J = 9.0 Hz), 7.70 (1H, d, J = 8.4 Hz), 7.60 (1H, d, J = 8.4 Hz), 2.75 (6H, s); ^{13}C NMR (151 MHz, CD_3OD) δ 167.5, 163.0, 140.4, 137.6, 136.8, 135.4, 131.0, 130.9, 130.8, 130.3, 129.8, 125.3, 124.6, 124.5, 123.5, 123.0, 118.2, 39.8; HRMS (ESI) Calcd for $C_{19}H_{19}N_6O^+$ ($[M-H(OSO_2Me)_2]^+$) 347.1615. Found 347.1617.
